# Synthesis and Modification of Polycarboxylate Superplasticizers—A Review

**DOI:** 10.3390/ma17051092

**Published:** 2024-02-27

**Authors:** Yuchen Xia, Wei Shi, Shuncheng Xiang, Xin Yang, Ming Yuan, Huan Zhou, Haotian Yu, Tingxiang Zheng, Jiake Zhang, Zhen Jiang, Liangjun Huang

**Affiliations:** 1 Hunan Provincial Engineering Technology Research Center for Novel and Carbon Neutral Road Material, Changsha University of Science and Technology, Changsha 410114, China; 19719886322@163.com (Y.X.); yuanming0214@163.com (M.Y.); zh1316193689@163.com (H.Z.); 15886738982@163.com (H.Y.);; 2 Science and Technology Affairs Center of Hunan Province, Changsha 410082, China; 15279301966@163.com; 3 College of Transportation Engineering, Tongji University, Shanghai 200092, China; zhjiake@tongji.edu.cn; 4 China West Construction Group Co., Ltd. Hunan Branch, Changsha 410082, China; jiangzhen1143@163.com; 5 Huaihua Dongxing Concrete Co., Ltd., Huaihua 418000, China; huang0408tx@yeah.net

**Keywords:** polycarboxylic superplasticizer, synthesis, cement paste, surface tension

## Abstract

The molecular-scale structural changes in polycarboxylic superplasticizer (PCE) can influence dispersion and water retention. Polycarboxylate superplasticizer, synthesized using different methods, may alter dispersion and water-reducing effects. The synthesis of PCE involves creating a novel macromolecular monomer with a controllable molecular mass, adjustable lipophilic, and hydrophilic moieties, as outlined in this study. This article reviews processes for synthesizing polycarboxylates and identifies the optimal method through orthogonal experiments to produce a modified polycarboxylate superplasticizer (PCE-P). The study investigated the effects of different PCE types and concentrations on the surface tension, fluidity, and ζ potential of cement paste. PCE-P, synthesized at room temperature, showed comparable performances in initial hydration and conversion rate in cement to PCE synthesized at high temperatures. PCE-P exhibited an increased slump but had a wider molecular weight distribution and longer main and side chains, leading to a 24.04% decrease in surface tension, indicating a good dispersibility.

## 1. Introduction

PCE was synthesized from unsaturated carboxylic acid monomers, long-chain alkane macromers, and other raw materials. It is a high-performance cement dispersant with good application prospects in concrete. PCE molecules encompass a variety of functional groups, such as -COOH, -SO_3_H, -NH_2_, and -OH groups. The hydration products of Portland cement (PC) induce the adherence of these groups to their surfaces, disrupting the flocculation structure between silicate particles and resulting in the formation of an adsorption layer. The spatial hindrance and electrostatic repulsion introduced by the added polycarboxylate superplasticizer have a significant impact on the interaction of cement particles at the water–solid activator interface. The uniform distribution of PC particles enhances flowability and other chemical properties [1,2,3,4]. Based on the form of the connection of its backbone and side chains, PCE can be roughly divided into two categories: polyester and polyether. Polyester-based PCE is characterized by the presence of ester bonds in both its main and side chains. The synthesis technology relies on the utilization of macromolecular monomers that undergo esterification or ester exchange reactions with methoxy polyethylene glycol acrylate (MPGA). Nevertheless, challenges persist in this process, encompassing difficulties in polymerization during esterification or ester exchange reactions, the potential for the self-polymerization of small monomers, and the occurrence of various side effects in the resulting product. Simultaneously, the polyether reaction is initiated through free radical polymerization, the direct polymerization of high-molecular-weight polymers, such as vinyl polyethylene glycol ether (carboxylic acid and sulfonic acid) and other small monomers in aqueous solution. The active group can react with the -COOH group on the main chain of polycarboxylates, and side chains with -C=C- double bonds can be linked to the main chain through free radical polymerization, which can change the structure and properties of polycarboxylates.

Compared with naphthalene superplasticizer, polycarboxylate superplasticizer has the characteristics of a low dosage and high water reduction rate. Its moisture reduction rate is 25–35%, with a dosage of about 1.5%, close to saturation, and a moisture reduction rate of 45–48% [1]. Meanwhile, polycarboxylate water-reducing agents have a good designability in molecular structure and environmental friendliness in synthesis. During the synthesis process, formaldehyde, concentrated sulfuric acid, or other toxic monomers cannot be used, and the damage to equipment, operators, and the environment is minimal [2,3]. In addition, polycarboxylate water-reducing agents have more synthetic materials and polymerization methods. Polymerization pathways include copolymerization, grafting polymerization, and block polymerization, etc., which are also relatively simple. In addition, PCE has a better stability, and we rarely find phenomena such as stratification or precipitation [4,5]. Finally, the polycarboxylate water-reducing agent has a good flowability and low slump loss for concrete. Compared with naphthalene superplasticizer, polyvinyl chloride has a large number of hydrophilic groups on its molecular chain, making it more adaptable to concrete [6].

Currently, significant research endeavors have been undertaken to enhance the structural design and synthesis methodologies of PCEs. Eltayeb et al. [7] exemplified this trend by synthesizing polycarboxylic acids through the utilization of acrylic acid and isopentenyl polyethylene glycol (IPEG) at a temperature of 60 degrees Celsius. Characterization of the synthesized compounds was accomplished employing 13C nuclear magnetic resonance (NMR) spectroscopy. The results indicate that, when the IPEG content is high, the molecular weight is around 40,000, and the molecular weight distribution is narrow, the dispersion effect of PCE is the best. In a separate investigation conducted by Ng et al. [8], an amide structure PCE was synthesized through the amidation reaction between amino methoxy polyethylene glycol (AMPG) and polyacrylic acid (PAA) at temperatures ranging from 130 to 150 °C. In the research conducted by Dalas et al. [9], the macromonomer employed was butyryl alkyl polyoxyethylene polyoxypropylene ether (BAPP), and 2,2-azobisisobutyronitrile served as the initiator. The reaction was initiated under a nitrogen atmosphere at 70 °C for 48 h, aiming to synthesize polycarboxylates with the capability to significantly expedite cement hydration. In the study led by Sahin et al. [10], acrylic acid and ω-methoxy polyethylene glycol methacrylate were utilized to synthesize non-adsorbed polycarboxylates within a 4 h timeframe at 80 °C. The results indicated that the synthesized polycarboxylate had no adsorption effect on cement and could significantly improve the dispersibility and flowability of cement. Researchers have successfully engineered PCEs with notable attributes, including a high water reduction rate and robust adaptability, among other exceptional performances. Concurrently, efforts have been directed towards broadening the spectrum of raw material sources and synthesis methodologies, minimizing production costs, and incrementally enhancing quality stability.

## 2. Synthesis of Macromolecular Monomers

The introduction of side chains into the molecular structure of PCE is achieved through the incorporation of other active monomers. The polymerization activity, molecular weight, and polarity of these active macromers are crucial factors influencing the quality and performance of PCE. The preparation method is outlined as follows:

### 2.1. Polymerization

Open-ring polymerization (ORP) is performed with unsaturated monomers like hydroxyalkyl acrylate or allyl alcohol as initiators, incorporating active hydrogen at the chain end. Then, ethylene oxide is added for polymerization to obtain active macromolecular monomers. Following a synthesis principle akin to polyethylene glycol monomethyl ether, its reaction is depicted in Equation (1) as follows:



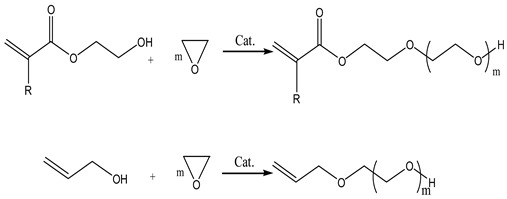

(1)


Plank et al. [11] employed methyl methacrylate (MMA) as the initiator, approximately 0.05% metal oxide complex as the catalyst, and incorporated effective polymerization inhibitors. They conducted ORP by combining epoxy propane and ethylene oxide, resulting in the synthesis of reactive macromers containing both components. The reaction was conducted at a temperature of 110 °C and a pressure of 0.3 MPa. In a high-pressure reactor, Chandel et al. [12] synthesized a range of macromolecular monomers with varying molecular weights. They employed allyl alcohol as the initiator, introduced ethylene oxide at 115–125 °C for polymerization, and sodium hydroxide as the catalyst. A series of PCEs were obtained by polymerizing the obtained product with maleic anhydride. Moreover, a novel synthesis method, termed chimeric ORP, has emerged for obtaining macromolecular monomers. This method involves utilizing MMA as the initiator and Mg-Al hydrotalcite as the catalyst through high-temperature melting. This mixture is then subjected to a continuous ring-opening reaction with ethylene oxide, becoming embedded into the ester bond in MMA. Generate novel active macromers produced through the reaction are illustrated in Equation (2):



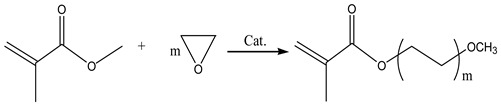

(2)


Abdolhosseini Qomi et al. [13] employed phenothiazine as an inhibitor to calcine magnesium aluminum hydrotalcite into magnesium aluminum oxide at 500 °C. This product was then utilized for the ORP of methacrylate and ethylene oxide. After 5 h at 150 °C, a new type of macromolecular monomer with a molecular weight of several hundred was obtained. In summary, the industrialization of this method is significantly hindered by its harsh conditions, substantial side effects, low conversion rate, and slow reaction speed.

### 2.2. Direct Esterification

The direct esterification of acrylic acid or maleic anhydride with polyethylene glycol monomethyl ether of varying molecular weights is the most common and traditional method for synthesizing active macromolecular monomers. This approach offers advantages such as low cost, a straightforward process, and ease of operation. The main factors affecting the reaction include type, time, temperature, structural ratio and amount of catalyst, and the equilibrium conversion rate of the reaction in Equation (3). Furthermore, the esterification reaction is reversible, necessitating the use of a water carrier to eliminate water and favorably shift the equilibrium towards the product direction [14].



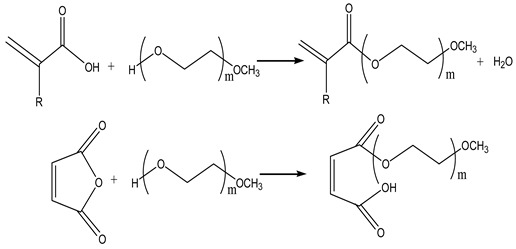

(3)


There are some examples. Poellmann et al. [15,16] synthesized PCE using polyethylene glycol as a raw material while using acrylamide, acrylic acid, methacrylic acid, methyl allyl sulfonate, and polyoxyethylene glycol acrylate (PEO) with different chain lengths as small monomers. The FTIR results revealed that the molecular structure of the modified polycarboxylate superplasticizer (PCE-P) comprised sulfonic acid, carboxylic acid, and polyepoxyethane vinyl groups, exhibiting a comb-like molecular structure. GPC also found that the chain length of PEO and the number of macromers affected the average molecular weight and distribution of PCE-P copolymers, thus determining the ability of PCE-P to disperse cement particles. After using PCE-P in concrete, the water reduction rate will reach 25%. In the investigation conducted by Keiji et al. [17], MPEG and acrylic acid were chosen for the preparation of macromolecular monomers through an esterification reaction. The study extensively explored the impacts of various reaction conditions on the esterification rate. The optimal process conditions were subsequently determined as follows: the molar ratio of AA was 3:1, toluene as a water carrier, phenol as an inhibitor, phenol as 0.8%, p-toluenesulfonic acid as a catalyst, and esterification at 120 °C for 8 h. In FITR, it was observed that macromolecular monomers featuring C=C double bonds and MPGA with extended polyoxyethylene chains could be generated.

The rate and extent of the esterification reaction are influenced by various factors, including temperature, duration, type and quantity of catalyst, and molecular weight of MPEG, among others. Consequently, these factors also impact the performance of PCE [18,19].

### 2.3. Acylation Reaction

The acylation process commences by combining methacrylic acid with chlorides, such as thionyl chloride, phosphorus trichloride, and phosphorus pentachloride. Subsequently, the introduction of polyethylene glycol monomethyl ether and a catalyst facilitates the generation of macromolecular monomers in the form of esters or amides. The chemical reaction is represented by Equation (4) below. Acylation is recognized as an irreversible and highly reactive procedure. However, a notable drawback lies in the poor stability of methacryloyl chloride, rendering it susceptible to decomposition and challenging to preserve for extended periods. Furthermore, its cost significantly surpasses that of corresponding carboxylic acids, imposing certain limitations on its industrial application.



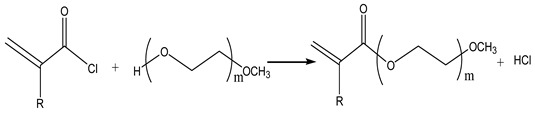

(4)


Ibrahim et al. [20] conducted a modification of MPEG by converting its terminal hydroxyl group into a mini hydroxyl group. Subsequently, methacryloyl chloride was employed as an acylating agent in the acyl chloride reaction, leading to the modification of MPEG and the preparation of active monomers featuring double bonds. An infrared spectrum analysis showed that the bonds of the main and side chains of the product transformed from traditional ester bonds to amide-imide bonds. At the same ionic strength, this copolymer also exhibited a larger hydrodynamic diameter with a higher water reduction rate at the same dose.

### 2.4. Ester Transfer

This method considers the preparation of macromolecular monomers by combining MMA and polyethylene glycol monomethyl ether under catalyst conditions. To improve the exchange rate of esters, small molecules generated in methanol and other processes should be continuously removed due to their reversibility. The reaction is Equation (5), as follows:



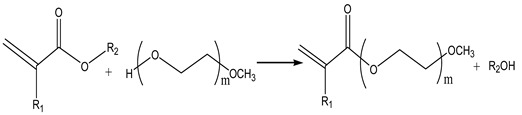

(5)


In the study of Winnefeld et al. [21], ethylene glycol monoethyl ether acetate was initially synthesized as an intermediate and subjected to an ester exchange reaction with methacrylic acid. Subsequently, a macromonomer of methacrylic acid polyethylene glycol monoester was prepared. The optimal reaction conditions encompassed a 7% tetra butyl titanate catalyst, 0.1% 2,2,6,6-tetramethylpiperidine oxide as an inhibitor, a reaction temperature of 130 °C, and a reaction time of 3 h, resulting in a macromolecular monomer conversion rate of up to 88.7%.



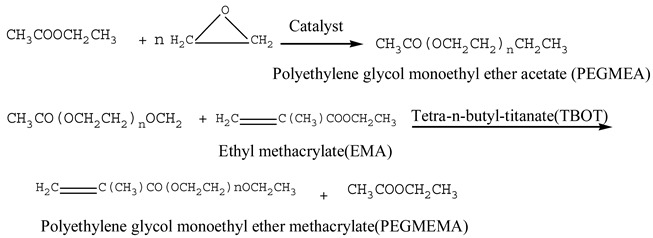

(6)


In infrared spectroscopy, the C=O bond of a 1717 cm^−1^ carboxyl group, the C-O-C bond of 1107 cm^−1^, and the C=C bond of 1636 cm^−1^ are easily discovered, indicating the presence of C=C double bonds and long polyoxyethylene chains in the product.

In the ester exchange study conducted by Flatt et al. [22], MMA and MPEG were employed as precursor materials, with phenothiazine serving as an inhibitor and sodium hydroxide as a catalyst. Methanol removal was conducted under reduced pressure at 50 °C, with a reaction duration of 3 h, resulting in an exceptional conversion rate of 99%. In the infrared spectrum, the emergence of the carboxyl group absorption peak, coupled with the disappearance of the hydroxyl group absorption peak, attests to a notably high esterification rate of the synthesized product.

Similarly, in the work by Gupta et al. [23], utilizing MMA and methoxy polyoxyethylene ether (MPEO, *n* = 23) as raw materials, a transesterification approach was applied. The optimization of catalysts, inhibitors, and synthesis conditions facilitated the successful synthesis of the macromonomer methoxy polymethyl methacrylate (MPEOMA), achieving an exemplary transesterification rate of approximately 98%.

### 2.5. Other Methods

Additionally, there is the direct aging method. In practical applications, methacrylic acid is commonly derived through the hydrolysis of the corresponding nitrile. Hence, substituting hydroxyl donors with alcohols allows for the direct synthesis of esters. The reaction is represented by Equation (7), as follows:



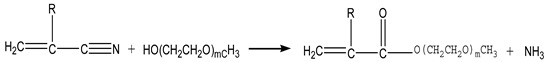

(7)


Polymerization uses alcohol and ethylene oxide. Olefin alcohols, instead of ethylene glycol alcohols, can directly polymerize with ethylene oxide to form double-bond terminated polyethylene glycol. The reaction Equation (8) is as follows:



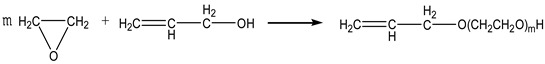

(8)


## 3. Synthesis of PCE

### 3.1. Direct Polymerization

In this method, the side chain of polyether is introduced into the main chain, which needs a polymerizable reactive macromonomer such as MPGA.

Şahin et al. [24,25] systematically investigated various monomers, ratios of different monomers, and adjusted reaction processes. A series of polycarboxylate superplasticizers (PCEs) was synthesized using allyl alcohol-polyethylene glycol (APEG, EO = 45), acrylic acid (AA), maleic anhydride (MAL), 2-acrylamido-2-methylpropane sulfonic acid (AMPS), and ammonium persulfate (APS) as raw materials. To determine the optimal process for PCE, extensive research was conducted on various reaction conditions, including reactant concentrations, temperatures, and monomer molecular ratios.

Jang et al. [26] fabricated two sets of comb-like copolymer dispersants through direct polymerization, featuring side chain lengths ranging from 8 to 48. Dessalle et al. [27] engineered a novel methacrylate polycarboxylate with a polyethylene glycol hydroxyl side chain at the end, deviating from the conventional methacrylate with a methoxy side chain. The outcomes indicated that the copolymer exhibited a comb structure and displayed excellent adaptability in cement, suggesting promising application prospects. Mastali et al. [28] conducted an analysis on the characteristics of the PEO side chain length and polymerization degree. The results showed that, under the condition of high w/c, the influence of the chemical structure on slurry flowability was not significant. Tran et al. [29] fabricated PCE in an aqueous solution, employing APEG, MAA, MA, and sodium methacrylate sulfonate (MAS) as monomers, with ammonium persulfate serving as the initiator. The optimal synthesis conditions were established as follows: maintaining a specific molar ratio, utilizing an initiator dosage equivalent to 5% of the monomer weight, and allowing a reaction time of 4 or 5 h. This formulation aimed to introduce anionic polar groups, such as -COOH and SO_3_H, into the PCE backbone, creating a hydrophilic structure characterized by robust hydrogen bonding in aqueous environments. This hydrophilic backbone is instrumental in forming a stable three-dimensional protective layer, providing steric hindrance. The modulation of functional group proportions in both the polymer main chain and side chain is believed to achieve structural balance, thereby enhancing the water-reducing properties [30].

Plank et al. [31] employed direct polymerization to synthesize a novel PCE, utilizing allyl polyethylene glycol, methyl acrylate, maleic anhydride, and ammonium persulfate in the polymerization process. The study systematically investigated the influence of monomer ratios, initiator quantities, reaction time, reaction temperature, and dropwise addition duration on the dispersion and water retention properties of the PCE. Through orthogonal experiments, the optimal synthesis conditions were determined. A comparative analysis of the performance of the synthesized product was conducted against another widely used product derived from MAA and MPEG. Yamada et al. [32] innovatively incorporated citric acid into the polycarboxylic molecule to synthesize an effective PCE, addressing challenges related to concrete slump loss, bleeding, and segregation. The optimal PCE formulation was achieved with a specific monomer ratio (n(MA):n(AA):n(SAS):n(PEGAA):n(PEGCM)) = 0.3:0.2:0.05:1:0.1), employing an initiator of 0.8% (NH_4_)_2_SO_4_ by the mass of vinyl monomers, a reaction time of 2 or 3 h, and a reaction temperature of 80 °C. The application results demonstrated a 32% water-reducing ratio, a five-hour extension in delay time, and concrete with PCE exhibited remarkable slump retention, absence of segregations.

### 3.2. Function Aggregation

Functional polymerization is viewed as a modification of the base polymer, typically involving the esterification of polyether at elevated temperatures and subsequent grafting into the main chain.

Gharanjig et al. [33] utilized PCE-grafted alkoxyimines as reactants in quantities ranging from 10% to 20% of -COOH moles. The blend underwent heating at 150 °C for 1.5−3 h, followed by the addition of a specific catalyst and cooling at 100–130 °C to attain the desired product. Felekoğlu et al. [34] combined a specific proportion of MAS, water, and ammonium persulfate in a three-necked flask equipped with a condenser and agitator. The mixture was heated to 80 °C for 3.5 h, yielding a yellow liquid. Subsequently, the PCE was blended with dimethyl sulfoxide at 110 °C and refluxed for 5 h, resulting in a novel PCE with a solid content of approximately 30%. The infrared spectra revealed peaks corresponding to -OH, -SO_3_H, -C=O, and C-O functionalities. The optimal performance of the graft copolymer was achieved when the molar ratio of polyethylene glycol, sulfonic acid, and side-chain carboxyl was 0.104:0.354:0.542.

Plank et al. [11] synthesized and tested many PCEs based on the molecular weight, density, and length of the branched chain. The results showed that the higher molar fraction was more adsorbed than the lower molecular fraction. In the polymerization of APEG with MA, Poplar synthesized PCE-1 with AIBA · 2HCl as the initiator. PCE-2 was synthesized from vinyl ethoxylated methyl ester (TPEG), AA, and methacrylate sulfonate (MAS) [35]. The results showed that the slump retention and compressive strength enhancement of concrete with PCE were better than those with PCE induced by ammonium persulfate. Silva et al. [36] prepared PCE from APEG, AA, maleic anhydride acid (MAD), and sodium methacrylamide (SMAS). PCE has good compatibility with cement. The prepared concrete had the advantages of a low slump loss and so on. When the content of the solid was 0.3%, the water reduction rate of concrete was 32.4%.

Masoudi Soltani et al. [37] formulated polycarboxylate superplasticizer (PCE) through the synthesis of poly (maleic anhydride) utilizing maleic anhydride, sodium methyl methacrylate sulfonate, and allyl-derived polyethylene glycol (PEG) as source materials, employing ammonium persulfate as the initiating agent. The optimal synthesis process conditions for polymaleic anhydride PCE were determined through cement paste fluidity testing. The optimal molar ratio of maleic anhydride, PEG, and sodium methyl methacrylate sulfonate was 4:1:0.2 and the optimal molecular weight of PEG was 2400. The initiator point was connected twice and polymerized at 80 °C for 5 h. In the exploration conducted by Ferrari et al. [38], a detailed examination of individual synthesis factors was undertaken to determine the optimal raw material ratio. This investigation encompassed the assessment of PCE performance and the characterization of its molecular structure. The test results attested to the successful incorporation of the targeted functional group into the structure. The GPC-measured data revealed the weight average number (Wn), molecular weight (Mw), side chain length (SCL), and main chain length (MCL) values for APEG- and TPEG-type PCE, demonstrating values of 103,000 g/mol, 14,700 g/mol, and 84,500 g/mol and 7540 g/mol, 2.5 nm and 1.8 nm, 15.3 nm and 15.3 nm, respectively.

Nevertheless, functional polymerization encounters certain challenges, including an increased difficulty in adjusting the composition and molecular weight. Along with challenges in esterification during practical applications, this remains a concern. Moreover, the ongoing generation of H_2_O can induce phase separation during the esterification reaction. Hence, the pivotal factor lies in the judicious selection of a polyether with the optimal compatibility.

### 3.3. Graft Polymerization

This approach primarily aims to address the drawback of functional polymerization, enabling the controlled regulation of the molecular weight of the polymer backbone to mitigate the compatibility issues between PCE and polyether. During the polymerization process, the introduction of side chains into the main chain is facilitated by utilizing polyether monomers containing -COOH groups. Otherwise, accomplishing this task would prove exceedingly challenging.

Schmid et al. [39] synthesized a novel PCE with an MMA backbone and a hydroxyl-terminated side chain. They successfully obtained branched-chain PCE, demonstrating an excellent adaptability to cement and robust early-stage performance. Uskoković [40] introduced acrylate monomers, chain transfer agents, and initiators into a solution containing MPG at 60 °C for 45 min. Subsequently, the system was heated to 120 °C, dehydrated under the protection of N_2_, and the catalyst was added at 165 °C and grafted for 1 h. Finally, he obtained a good dispersion, low slump loss, and low lead gas performance of the new asphalt. Afroughsabet et al. [41] carried out a series of polymerization experiments in an attempt to find out the relationship between the synthesis conditions and the dispersion of cement pastes. The reaction temperature, initiator (APS), and reaction time were 70 °C, 0.5%, and 9 h, respectively.

Gautam et al. [42,43] conducted the synthesis of a comb-like PCE using MPEOMA, AA, MA, and sodium allyl sulfonate (SAS) through aqueous copolymerization initiated by ammonium persulfate. The findings indicated that the resulting PCE exhibited an exceptional dispersing ability and favorable compatibility with different types of cements. When the cement content was 0.3% and the water-cement ratio was 0.26, the fluidity of cement paste could reach 265 mm. A range of polyether-based PCEs were synthesized through free radical polymerization in an aqueous solution, utilizing sodium vinyl sulfonate as monomers, allyl polyethylene glycol (AEO), and maleic anhydride. The effect of synthetic processes on the properties was studied. The findings indicated that optimal conditions were achieved with a mass ratio of 3:5 for allyl polyethylene glycol (AEO) to maleic anhydride, an initiator quantity of 6–7%, and a reaction temperature ranging between 75 and 85 °C.

### 3.4. Free Radical Polymerization

Traditional radical polymerization is uncontrollable because the chain it grows in has a free radical active center with a strong double-termination trend called coupling or dissimulation. However, scientists have developed a new method of polymerization that can be controlled, called reversible addition-fracture transfer (RAFT). In conventional radical polymerization systems with Azobisisobutyronitrile, Benzoyl peroxide, or ammonium persulfate as initiators, we can add a large amount of chain transfer agent to make it controllable and obtain PCE with a small molecular weight distribution.

Water-soluble PCE was synthesized in radical polymerization at 75 °C by Shawl et al. [44]. The PCE synthesized for this investigation incorporated extended comb-like side chains and ionic groups, imparting both spatial effects and electrostatic repulsion. Hirata et al. used MPEGMA, MAA, and MMA to polymerize at 80 °C for 5 h. So, he made PCE with different molecular weights. 

Büyükyağcı et al. [45] synthesized a series of allyl polyethylene glycol-based PCEs via radical copolymerization in an aqueous solution. GPC was used to measure the molecular weight of the copolymer and the conversion of allyl polyethylene glycol. The optimal reaction conditions were as follows: n(APEG2400):n(MA):n(AM) =1.0:4.0:1.5, an initiator dosage of 3.5% (mol), a reaction concentration of 60% (mas), a reaction temperature of 65 °C, and an acrylamide dropping time of 8 h. The average molecular weight was about 43,800 and the monomer conversion rate was 91.5%. The loss was small. Simultaneously, its performance in concrete applications was commendable, achieving a water reduction rate of 30%. In the work of Cho et al. [46], PCE was synthesized through the free radical copolymerization (FRC) of dendritic-activated macromonomer and AA in water. The optimized reaction conditions were as follows: hydrogen peroxide and l-ascorbic acid (1-AA) served as initiators, with 1-AA and chain transfer agent dosages set at 1% and 2% of the monomer, respectively. The molar ratio of AA to the macromolecule monomer was maintained at 4:1, and the polymerization concentration was set at 50%.

Polyethylene glycol monomethyl ether methacrylate (MPEGMAA) was prepared by Güneyisi et al. [47,48] for the synthesis of PCE. The effects of different methods of water introduction, multi-component inhibitors, and amount of supported catalyst on the esterification rate of MPEGMAA were studied, the macromonomer was prepared successfully, the esterification rate was over 99%, and the storage stability of the macromonomer was studied. The results showed that, in the redox system, adding 0.25–0.3% pure copolymer, the w/C was 0.29, the temperature could be reduced to 50–60 °C, the water reduction rate was more than 28%, and the fluidity of slurry was between 250 mm and 260 mm, with a liquidity retention rate of more than 95%.

Hirata et al. [49] mixed small functional monomers of MA and acrylamide (AM) with APEG or pentenyl polyoxyethylene ether (TPEG) to synthesize two different types of PCE. By discussing the factors of polymerization, the optimum technological parameters of synthesizing PCE were obtained: the optimum molar ratios of synthesizing PCE were n(APEG):n(MA):n(AM) = 1:1.8:1.2 and n(TPEG):n, respectively. Adams [50] modified PCE with hydroxypropyl acrylate (HPA) and evaluated the dispersing ability, dispersing retention ability, and rheological properties of the modified PCE. The results showed that the carboxyl density was the most important factor affecting the initial dispersion of PCEs. The higher the concentration of carboxyl, the higher the initial dispersion performance. When the carboxyl density is greater than 7:1, the initial dispersion performance will not change significantly.

### 3.5. Synthetic Conditions

In contrast to the high-temperature synthesis of PCE, the synthesis of PCE-P involves the direct polymerization of polymerizable monomers. Initially, an oxidizer is introduced into the system, succeeded by the addition of a reducing substance. This process utilizes the heat and free radicals generated by the redox system to initiate and sustain the entire polymerization.

#### 3.5.1. Initiating System with Vc as Reducing Agent

Vc, which has a strong reducibility and special structure, is the main reductant in the synthesis of PCE. With oxidizing substances, it is easy to generate heat and free radicals, so we can use it to synthesize PCE. In addition, the reaction system consists of Vc and hydrogen peroxide.

Lee et al. [51] conducted a series of experiments aiming to synthesize PCE, and they identified the optimal reaction conditions. The redox initiator system with a H_2_O_2_:Vc ratio of 4:1, along with 1.5% hydrogen peroxide (relative to the macromonomer mass), 1.2% sodium phosphate (relative to the macromonomer mass, serving as a chain transfer agent), and 6% sodium methallyl sulfonate (SMAS, relative to the macromonomer mass), proved effective in producing a high concentration (80%) of high-performance PCE. The results showed that the flowability of paste could reach 285 mm, 288 mm in 60 min, and 282 mm in 120 min when the dosage of PCE was 0.20%. Simultaneously, the concrete application performance of this PCE was noteworthy, achieving a water reduction rate of 30%. Gelardi et al. [52,53] employed FRC of dendritic-activated macromonomer and acrylic acid in water to synthesize PCE. The optimal reaction conditions involved the use of 1-AA and hydrogen peroxide as initiators, with the dosage of 1-AA and the chain transfer agent set at 1% and 2% of the monomer, respectively. The molar ratio of AA to the macromolecule monomer was 4:1 and the polymerization concentration was maintained at 50%.

The results of GPC showed that the average molecular weight of PCE was 47,500 and the conversion of DAM was about 89.6%. Fu et al. [54] employed 2-dimethylallyl polyoxyethylene ether (HPEG), AA, and MA as monomers, along with a composite initiator system comprising ammonium persulfate (APS) and Vc hydrogen peroxide (V_C_-H_2_O_2_). They introduced a new type of PCE synthesis using thioglycolic acid (TGA) as a chain transfer agent. The optimum conditions of free radical polymerization were as follows: a mass ratio of HPEG:AA = 8.5:1.0 and composition of initiator (V_C_-H_2_O_2_):APS = 0.9:1.0, H_2_O:Vc = 5.5:1.0. The quantity of MA, the total initiator, and TGA were 1.5%, 1.2%, and 0.4% of the mass of HPEG, respectively. The reaction was carried out at room temperature (20–40 °C) for a duration of 3 h. Outstanding PCE demonstrated a notable water-reducing ratio of 33.1%, along with effective slump retention and dispersion capabilities. Rymeš et al. [55,56] similarly employed this initiation system in their patents to synthesize PCE at room temperature. This method offers several advantages, including the absence of an external heat source requirement, a high solid content of PCE, and cost-effectiveness in transportation.

#### 3.5.2. An Initiating System That Uses Other Agents as Reducing Agents

Scientists have explored alternative initiator systems utilizing reagents beyond Vc as reducing agents. Careful consideration of factors such as the structure of the reducing substance, its electromotive force, minimum activation energy, and the structure of the free radical is essential in these systems.

Guan et al. [57] developed a reaction system where the mass ratio of hydrogen peroxide to formaldehyde was 1:1 and the total content of sulfoxide ester was controlled at 2.5% of the monomer mass in the novel reaction system. Thus, a high-performance PCE was obtained. Sahin et al. [10] employed a low-temperature synthesis method (15–20 °C) to produce PCE, yielding properties comparable to those synthesized at 70 °C. This approach not only reduced steam consumption, but also saved energy, contributing to a reduction in production costs. Wang et al. [58] obtained a new colorless PCE by adding acrylic acid, sodium methacrylate, and chain transfer agent with ammonium persulfate as an initiator for 3 h and adjusting the PH to 5. The results showed that the synthesized PCE had a certain water reduction rate and good slump control ability, with no loss in one hour and less loss after two hours. Based on orthogonal experiments, Maruyama et al. [59] conducted the synthesis of PCE through radical polymerization involving allyl alcohol Polyethylene glycol and other small-molecule monomers at low temperatures. The optimal polymerization conditions were as follows: the n (MA):n (APEG):n(AM):n(AA) molar ratio was 1.6:1.5:1.5:4.0 and the initiator dosage was sodium dithionite 4%, H_2_O_2_ 4%. The resulting product exhibited a high water reduction rate and excellent slump-holding capacity, contributing significantly to the strength enhancement of concrete.

## 4. Application

**Figure 1:**
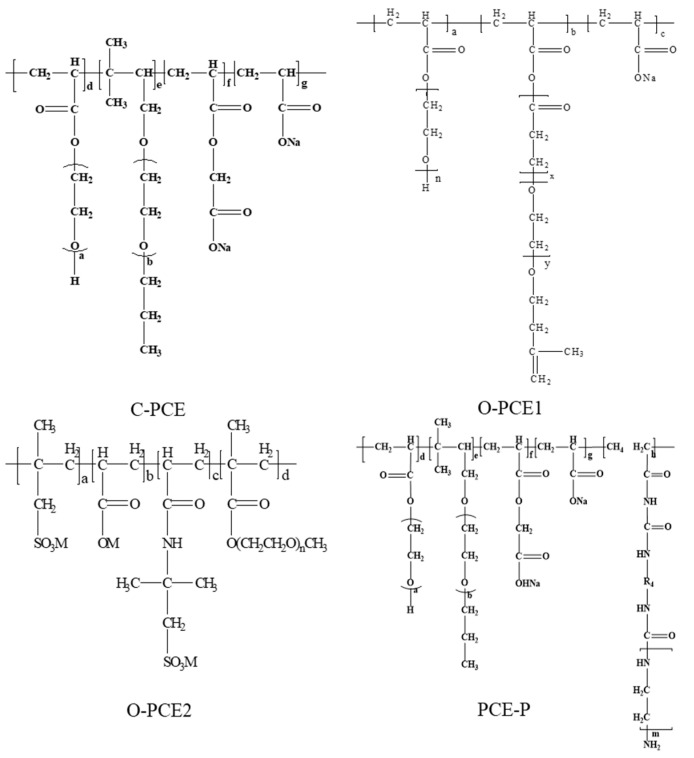
Chemical formula of different PCE.

Limited research has been conducted on the adsorption and dispersion properties of PC when combined with various polycarboxylate superplasticizers. PCE-P with a consistent main chain length but varying side chain lengths was synthesized by controlling the molar ratio of macromonomer to methacrylic acid. At the same time, we selected our own synthesized ordinary polycarboxylate superplasticizer (O-PCE1 and O-PCE2) and commercially available polycarboxylate superplasticizer (C-PCE), with molecular structures shown in Figure 1, to test the surface tension and its effect on the fluidity of cement paste. The surface tension and its effect on the fluidity of cement paste were also tested, which provides a theoretical basis for the design, synthesis, and selection of polycarboxylate superplasticizer. The relevant chemical components of cement (P·I42.5, Changsha Hetian Baishi Building Materials Co., Ltd., Changsha, China) are shown in Table 1.

### 4.1. Surface Tension

The A-60 automatic surface tension meter (American Cono Industries Co., Ltd., Boston, MA, USA) is used to measure the surface tension of the PCE in different doses. For the testing of surface tension between the liquid and solid phases of cement pastes, we used a beaker containing 50 g of PC and a set water–cement ratio of 0.4. Beakers were employed to mix PCE with 20 g of water at varying concentrations (0%, 0.1%, 0.2%, 0.3%, 0.4%, and 0.5% of the total mass). First, the liquid was mixed slowly for 2 min, then mixed vigorously for 1 min. Afterwards, the cement mixture obtained was subjected to centrifugation, and the surface tension of the resulting supernatant was subsequently measured.

**Figure 2:**
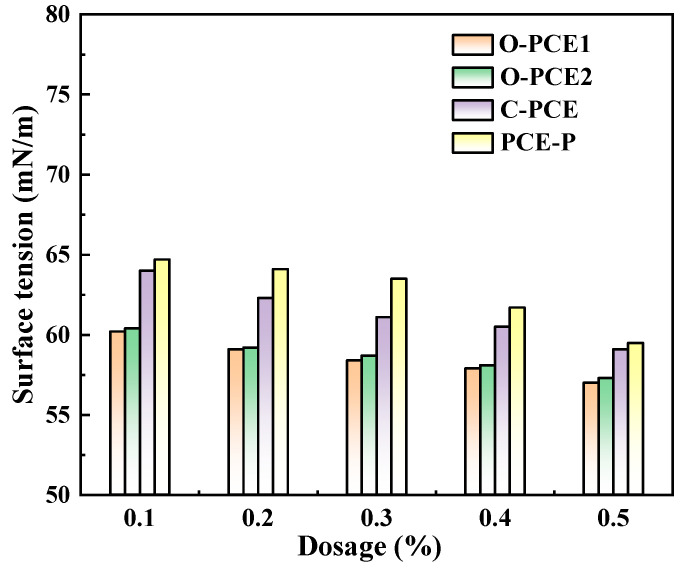
Surface tension of fresh cement pastes.

Through experimental analysis [60], we selected the optimal water–cement ratio of 0.4 as the experimental water–cement ratio. Figure 2 below shows the effect of PCE-P on the surface tension of fresh cement pastes.

From Figure 1, the incorporation of different PCE types led to a reduction in the surface tension of the cement paste. The surface tension decreased further with an increase in PCE content, and the increase in PCE-P was the most obvious. This can be attributed to the compact and high conversion of monomers, along with the molecular weight distribution of the PCE-P produced by the REDOX system using hydrogen peroxide and ammonium persulfate Vc. This led to an outstanding overall performance, characterized by elongated side chains in comparison to traditional PCE, resulting in enhanced steric hindrance and dispersion.

Moreover, the concentrations of Ca^2+^, Na^+^, and OH^-^ ions in the liquid phase decreased as a result of the reduced solubility of the mixture following the addition of various PCEs. In the initial stages of PC hydration, the Si-O bond underwent cleavage within an alkaline environment. It then bound with hydrated and dissociated ions to create a C-S-H gel. As a consequence, the concentration of surface-active particles in the Portland cement (PC) decreased, thereby influencing the surface tension of the liquid phase [61,62].

### 4.2. Flowability

Based on the GB/T8077-2012 standard [63], the initial flowability, as well as the net paste flowability at 1 h and 2 h, were assessed using the hollow column model test; the best material quantity obtained through team experiments was 300 g of PC, a water–cement ratio fixed at 0.29, and PCE quantity was calculated by total mass [64,65].

The diagram shows that the fluidity of fresh cement pastes changes with the type and amount of PCE. The initial flowability of cement paste mixed with three kinds of PCE increased continuously with the increase in content, and the maximum increase was 342.8%. The rise in fluidity could account for the escalation in PC adsorption capacity with the augmentation of PCE content, thereby reinforcing the system’s free water absorption capability and fluidity. In addition, the effects of various mineral admixtures (such as silica fume, fly ash, and mineral powder, etc.) on the adsorption and rheological properties of cement have been tested in previous studies, with the results showing that an increase in SCM content will lead to the continuous decline in cement rheological properties [66,67,68,69,70,71].

### 4.3. Zeta Potential

**Figure 3:**
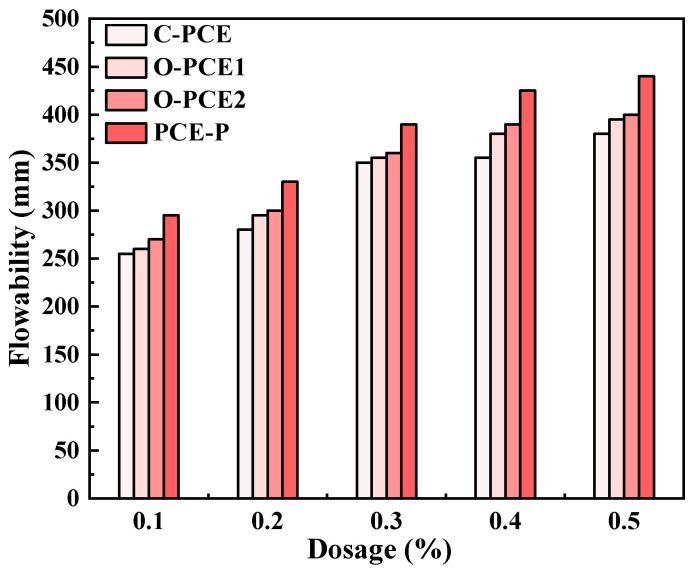
Fluidity of fresh cement pastes.

The ζ potential of the cement pastes mixed with PCE was studied by varying the level of the superplasticizer content (0%, 0.1%, 0.2%, 0.3%, 0.4%, and 0.5%) while maintaining a constant water–cement ratio of 0.4 [72]. As shown in Figure 3, the introduction of various PCE types negatively impacted the dispersibility of PC, with a diminishing effect observed in the order of PCE-P, O-PCE2, O-PCE1, and C-PCE.

**Figure 4:**
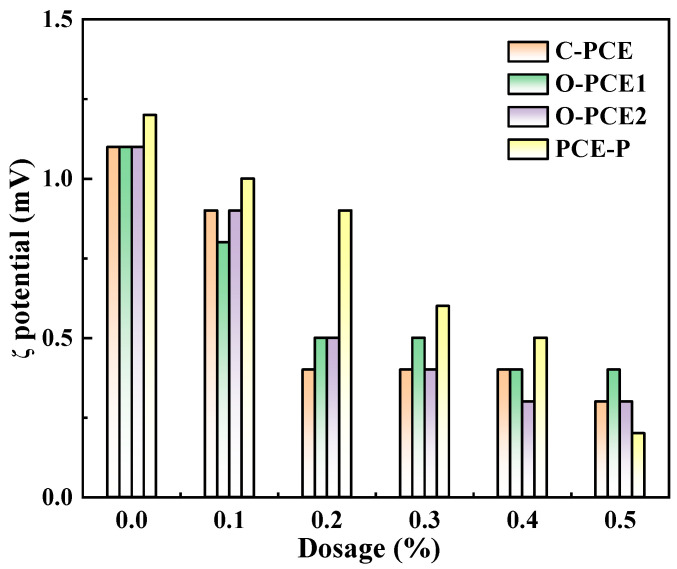
The ζ potential of fresh cement pastes.

Figure 4 shows the effect of four different water-reducing agents on the potential of fresh cement, we can see from the picture as the amount of water-reducing agent increased, the potential gradually decreased. During PC hydration, the surface potential of particles changes and the potential of the diffusion layer undergoes corresponding changes. Preserving electrostatic repulsion is crucial for sustaining the ζ potential. The presence of PCE on cement particles’ surfaces leads to the elongation of the functional agglomerated formaldehyde chain into the solution. This leads to the establishment of hydrogen bonds with H_2_O molecules in pure PC pastes. The creation of solvent hydrate shells induces lubrication and steric hindrance effects [73,74]. These roles represent the primary functions of PCEs. Moreover, the mixed PCEs contain numerous anionic groups that influence the charge distribution of the Stern bipolar layer on the local surface of the PC particles. A notable distinction exists between the ion concentrations on the surface of the PC particles and the predominant ion concentrations in the slurry. The accumulation of anions near the surface layer shields the surface charge, consequently reducing the ζ potential. Hence, the ζ potential decreases with an increase in PCE-P content.

Furthermore, the presence of polycarboxylic superplasticizer led to an increase in the hydration products of tricalcium aluminate (C_3_A) and tetracalcium aluminoferrite (C_4_AF) in the PC. Positively charged particles resulted in a positive ζ potential [20,75]. This elucidates the alteration in the ζ potential. The hydration products of C_3_A and C_4_AF in the PC increased with an increase in the dosage of polycarboxylic superplasticizers, and the ζ potential became positive as they become positively charged [76,77]. The main minerals that made up the PC were found to be negatively charged particles in dicalcium silicate (C_2_S) and calcium silicate (C_3_S) hydrated pastes and positively charged particles in C_3_A and C_4_AF. Silicates are less soluble than aluminates. Thus, the ζ potential showed a positive value because positively charged hydrate products dominate [78]. Despite the addition of a specific quantity of anionic polycarboxylic acid-based superplasticizer, there was a declining trend observed in the ζ potential on the surface of the cement particles.

## 5. Conclusions

(1) The properties of PCE are significantly influenced by the structural characteristics of the macromonomer. Therefore, the design and synthesis of PCE should initiate from the macromonomer, focusing on controlling the molecular weight and adjusting the proportions of hydrophilic and lipophilic groups. A macromonomer with a reasonable structure and stable performance should be prepared, and the existing polyether and polyester should be modified using block and grafting.
(2) The higher the carboxyl content of the PCE main chain, the more suitable the length of the side chain. The length of the main chain and side chain and molecular weight have more influence on the dispersion of PCE in cement. Therefore, the longer the length of the main chain and side chain, the better the dispersion of PCE. The dispersion of PCE in cement cannot be adequately explained by a singular theory. Multiple factors, including electrostatic repulsion, steric hindrance, the chain length of the main chain or side chain, and molecular form, among others, should be taken into consideration to provide a comprehensive understanding.
(3) In contrast to other polycarboxylate superplasticizer such as sodium bisulfite and tonalite, polycarboxylate molecules generated through the REDOX system of hydrogen peroxide and ammonium persulfate Vc exhibit a compact high monomer conversion and molecular weight distribution. The outcome is an outstanding overall performance, characterized by extended side chains in comparison to conventional PCE, leading to enhanced steric hindrance and dispersion. Under the same experimental conditions, it was found that the dispersibility of polycarboxylate increased with the increase in the AA/TPEG mole ratio.
(4) With an increase in the content of polycarboxylic superplasticizer, the surface tension of cement paste decreases. The reduction in surface tension results in a decline in the stability of cement paste and a decrease in ζ potential.

## Figures and Tables

**Table 1:** Chemical composition of cement (%).

Material	SiO_2_	Al_2_O_3_	Fe_2_O_3_	CaO	MgO	SO_3_	TiO_2_	K_2_O	Na_2_O	Total	Loss
PI 42.5	21.18	4.73	3.41	62.49	2.53	2.83	-	-	0.56	97.73	1.76

## Data Availability

Dataset available on request from the authors.
